# Adverse events identified by a trigger tool as indicators of patient safety and safety management in a medical department

**DOI:** 10.3389/fpubh.2025.1711864

**Published:** 2025-12-17

**Authors:** Ludmila Pierdevara, Ana Maria Porcel-Gálvez, Margarida Eiras

**Affiliations:** 1 Escuela Internacional de Doctorado de la Universidad de Sevilla, Seville, Spain; 2 Unidade Local de Saúde do Algarve, Departamento de Medicina – UHP/UHL, Faro, Portugal; 3 Departamento de Enfermeria, Universidad de Sevilla, Seville, Spain; 4 Instituto Politecnico de Lisboa Escola Superior de Tecnologia da Saude de Lisboa, Lisbon, Portugal

**Keywords:** global trigger tool, medical errors, patient safety, adverse events, safety management

## Abstract

**Background:**

Adverse events (AEs) in hospital settings pose a significant threat to patient safety, particularly in patients with multiple comorbidities. Systematic detection tools, such as the Global Trigger Tool, have proven to be more sensitive than traditional voluntary reporting systems in identifying AEs.

**Objective:**

This study analyzes the prevalence, characteristics, and factors associated with the occurrence of AEs in an internal medicine department, focusing on patients with multiple morbidities, using a combined approach that integrates the European Portuguese version of the Global Trigger Tool (GTT-PT) and a voluntary reporting system.

**Methods:**

This observational, retrospective, exploratory study was conducted in four internal medicine departments at a Portuguese hospital center. A total of 360 randomly selected clinical records were included in this study. AEs were identified using the GTT-PT and by analyzing voluntary incident reports. Sociodemographic and clinical variables were analyzed using binary logistic regression.

**Results:**

A total of 718 AEs were identified, of which 564 (78.6%) occurred during hospitalization. Most events were of moderate severity (categories E and F in the Institute for Healthcare Improvement classification). Length of hospital stay was the main predictor of AEs occurrence (odds ratio [OR] range 1.011–1.173). The use of a nasogastric tube was also significantly associated with increased AE risk (OR = 6.693). The GTT-PT detected significantly more events than the voluntary reporting system.

**Conclusion:**

The combined use of the GTT-PT and voluntary reporting systems is highly effective in detecting AEs in internal medicine settings. Length of hospital stay and use of invasive devices are key factors associated with AE occurrence. These findings underscore the importance of institutional policies that support a non-punitive safety culture and encourage the systematic integration of proactive monitoring methodologies into clinical practice.

## 1 Introduction

Patient safety is a cornerstone of modern healthcare and presents a complex multidimensional challenge for healthcare systems worldwide. Adverse events (AEs), defined as unintentional injuries or complications arising from healthcare interventions rather than from underlying diseases, remain a leading threat to the safety of hospitalized patients. AEs have a direct impact on the physical and psychological well-being of patients and place significant financial strain on healthcare services, contributing to increased morbidity, mortality, and the overall economic burden of hospitalization (1, 2).

International studies have shown that AEs occur in approximately 10% of hospital admissions, and approximately 50% of these cases are preventable through safer clinical practices and more effective prevention systems (3). Incidence rates depend on the clinical context, medical specialty, patient profile, and AE detection method used. This variability underlines the need for consistent and sensitive tools that permit a more accurate identification of the true impact of AEs on routine hospital practice.

Voluntary reporting and trigger tools are the two most used methods for detecting AEs. Voluntary reporting systems, while valuable for fostering a safety-oriented and institutional culture, have significant limitations: they depend on the individual initiative of healthcare professionals, are influenced by cultural and behavioral factors, and often underreport common events, such as adverse drug reactions or failures in clinical monitoring (4). Trigger tools offer a more proactive and systematic approach by conducting retrospective reviews of clinical records to identify indirect indicators of potential AEs, such as sudden changes in laboratory values, the administration of antidotes, or interruptions in medication (5, 6).

The Global Trigger Tool (GTT), developed by the Institute for Healthcare Improvement (IHI), is one of the most widely used and studied tools in the field. The GTT uses a two-step review process conducted by multidisciplinary teams to identify AEs from a random sample of clinical records. Its effectiveness stems from its ability to detect a significantly higher number of events compared to voluntary reporting systems. Some studies have shown that the GTT can detect up to eightfold more events than those voluntarily reported by healthcare professionals (7). This characteristic establishes the GTT as a valuable tool for auditing and continuous quality improvement.

However, the practical application of the GTT and other trigger tools faces considerable challenges, including time demands, the need for qualified personnel and training, and adaptation of triggers to the clinical and cultural contexts of each country or institution (8). To address these limitations, several countries have developed national adaptations of the GTT. In Portugal, the GTT was translated, culturally adapted, and validated as the Portuguese version (GTT-PT), demonstrating promising reliability and applicability within the Portuguese hospital system (9). This adaptation represents a significant advancement toward aligning national practices with international patient safety standards; however, its practical implementation continues to face institutional and operational barriers.

One of the most critical areas of research on AEs includes patients with multiple morbidities, a population involved in an increasing proportion of admissions to internal medicine wards who are at particularly high risk of experiencing AEs. The clinical complexity associated with multiple morbidities, intricate therapeutic regimens, polypharmacy, physical frailty, and potential drug interactions render these patients significantly more susceptible to various forms of iatrogenic harm (10, 11). Moreover, managing patients with multiple morbidities in a hospital setting requires improved coordination among healthcare services, more frequent clinical evaluations, and complex decision-making, often conducted under time constraints. Several studies have associated this population with higher rates of adverse drug events, hospital-acquired infections, diagnostic errors, and inpatient falls (12, 13). Despite this evidence, most research on AEs has focused on general or specific populations, such as surgical or pediatric patients, leaving a gap in understanding the characteristics and incidence of AEs within multiple morbidity contexts (14, 15).

In Portugal, the systematic use of these tools remains limited to pilot projects or academic contexts, despite ongoing efforts to integrate the GTT-PT into quality evaluation routines in healthcare. A lack of reporting in voluntary systems remains a significant barrier, reflecting that the institutional culture is still influenced by fear of disciplinary consequences, insufficient constructive feedback, and absence of a non-punitive approach to errors. Recent studies have shown that fear of sanctions and perceptions of reporting are unnecessary, and that limited leadership support substantially reduces healthcare professionals’ willingness to report AEs. In addition, cultural factors, such as time constraints, lack of knowledge of reporting procedures, and workload pressure, have consistently been identified as obstacles to active participation in reporting systems (16–19).

Moreover, population aging and the increasing burden of multimorbidity highlight the urgent need for more effective strategies to monitor and prevent AEs. The World Health Organization has long advocated for the strengthening of hospital reporting and learning systems based on reliable and representative data (20). Recent studies further confirmed that early detection of AEs, combined with organizational learning, plays a critical role in reducing preventable harm and fostering a culture of continuous improvement (21, 22).

In this context, it is essential to investigate the performance of AE detection tools in real clinical settings, particularly within internal medicine departments, where case complexity is high and resources often limited. Few national or international studies have conducted in-depth comparisons of the combined use of trigger tools and voluntary reporting systems, particularly in populations with multiple morbidities. Understanding AE patterns in this population and evaluating the sensitivity and specificity of each method can provide robust evidence for the development of more effective safety policies adapted to local contexts.

This study aimed to analyze the incidence and characteristics of AEs in patients admitted to the internal medicine departments of two Portuguese hospitals, with a particular focus on those with multiple morbidities. To this end, a combined methodological method was used, including the GTT-PT and a voluntary AE reporting system, to identify factors associated with the occurrence of AEs, compare the detection capacity of the two tools, and explore their limitations and complementarities within the studied hospital setting.

In addition to advancing our understanding of AE epidemiology in Portugal, this study aimed to offer practical recommendations for implementing patient safety surveillance systems in hospital settings. This underscores the critical role clinical teams play in fostering a culture of quality, safety, and accountability. The insights generated may prove valuable to hospital administrators, policymakers, and researchers seeking to develop sustainable evidence-based interventions to improve healthcare safety, particularly in the face of evolving demographic and clinical challenges that demand more integrated and adaptive approaches. The use of internationally recognized methodologies, such as the GTT, combined with context-specific national data, allows the identification of priority areas for local improvement. Moreover, it supports structured comparisons of healthcare systems in other countries, facilitates international benchmarking, and contributes to strategic alignment with global quality and patient safety standards.

## 2 Materials and methods

### 2.1 Study design

This was a quantitative, observational, descriptive, exploratory, and cross-sectional study based on retrospective data analysis. The structure and reporting of this study followed the Strengthening the Reporting of Observational Studies in Epidemiology (STROBE) guideline to ensure transparency and methodological rigor in the scientific reporting process (23).

### 2.2 Context, population, and sampling

This study was conducted at the Department of Medicine of a university hospital center in southern Portugal, which includes four inpatient wards distributed across two hospitals that are part of the Algarve University Hospital Center (CHUA). This hospital center provides care to the population residing in the Barlavento Algarvio region, which includes a broad geographical area with diverse demographic and epidemiological characteristics. The internal medicine wards included in the study had a total capacity of 130 inpatient beds and were responsible for the clinical management of patients with a high burden of comorbidities, therapeutic complexity, and prolonged care needs. Participants were selected through simple random sampling in accordance with the methodological recommendations of the IHI for the use of the GTT. Randomization was conducted using a computerized random number generator to guarantee impartiality in the selection of clinical records. During each month of the study period, 12 clinical records were randomly selected from each inpatient ward. Of these, 10 were actually included for review, while the remaining 2 served as substitutes, being used only when the initially selected records did not meet the inclusion criteria or contained incomplete or illegible data. This approach ensured consistency in the number of records reviewed per month and reduced the potential selection bias associated with missing or poor-quality documentation.

### 2.3 Inclusion and exclusion criteria

The inclusion criteria were clinical records of patients hospitalized for more than 24 h, provided that the medical, nursing, and administrative discharge processes had been fully completed at least 2 months prior to sample selection. This time window was set to ensure completeness of the available records and full access to clinical documentation.

The exclusion criteria were the clinical records of patients diagnosed with acute mental illness during the analyzed hospitalization period because of the additional complexity in detecting and classifying AEs in these cases. Illegible records, particularly those with incomplete data or those unavailable in a searchable format, were also excluded.

### 2.4 Data sources and data collection instruments

Data were collected from three complementary sources to ensure a comprehensive assessment of AE occurrences in a hospital setting:

1. Electronic clinical records included medical, nursing, laboratory, and electronic prescription information related to the selected hospitalization periods. Information on patients’ medical history, diagnoses, prescriptions, diagnostic tests, clinical progression, therapeutic interventions, length of stay, and hospital outcomes was extracted. These records were the primary basis for the application of the GTT-PT tool and retrospective analysis of AE occurrence.
2. The voluntary incident and AE reporting system involved analyzing reports voluntarily submitted by healthcare professionals during hospital admissions included in the study. The reports were retrieved from the incident and AE reporting systems of the institution.
3. The GTT-PT was applied by a trained review team using a standardized methodology to identify potential AEs based on the presence of clinical triggers in patient records. This tool uses a two-stage review process: an initial screening conducted by nurses, followed by medical validation of positive cases and categorization of AE severity.

The GTT-PT is a validated instrument that has been culturally adapted for use in Portuguese hospitals. It offers a structured methodology for retrospective medical record reviews based on identifying predefined clinical triggers. The tool is organized into five modules covering distinct areas of hospital care: general care, surgical care, laboratory parameters and medication, intensive care, and urgent/emergency care. Each module contains a specific set of triggers that, when present, are identified in a medical record and prompt further analysis to determine whether an AE has occurred. The complete list of the 48 triggers used in the GTT-PT is presented in Table 1.

**Table 1:** List of triggers used in the GTT-PT tool.

Trigger code	Trigger description
Cares module triggers	
C1	Transfusion or use of blood products
C2	Code/arrest/rapid response team
C3	Acute dialysis
C4	Positive blood culture
C5	X-ray or Doppler studies for emboli or DVT
C6	Decrease of greater than 25% in hemoglobin or hematocrit
C7	Patient fall
C8	Pressure ulcers
C9	Readmission within 30 days
C10	Restraint use
C11	Healthcare-associated infection
C12	In-hospital stroke
C13	Transfer to higher level of care
C14	Any procedure complication
C15	Sleep disorders
C16	Skin lesions/macerations
C17	Other
Surgical module triggers	
S1	Return to surgery
S2	Change in procedure
S3	Admission to intensive care post-op
S4	Intubation/reintubation /BiPap in Post Anesthesia care unit (PACU)
S5	X-ray intra-op or in PACU
S6	Intra-op or post-op death
S7	Mechanical ventilation greater 24 h post-op
S8	Intra-op epinephrine, norepinephrine, naloxone, or flumazenil
S9	Post-op troponin level greater than 1.5 ng/mL
S10	Injury, repair, or removal of organ
S11	Any operative complication
S12	Other
Medication module triggers	
M1	*Clostridium difficile* positive stool
M2	Partial thromboplastin time greater than 100 s
M3	International Normalized Ratio (INR) greater than 6
M4	Glucose less than 50 mg/dL
M5	Rising BUN or serum creatinine greater than 2 times baseline
M6	Vitamin K administration
M7	Diphenhydramine, Livocet (Dichloroiodate de Cetirizine), Hydroxyzine use
M8	Flumazenil use
M9	Naloxone use
M10	Anti-emetic use
M11	Over-sedation/hypotension
M12	Abrupt medication stops
M13	SOS medication (Pain/ hypertension)
M14	Other
Intensive care module triggers	
I1	Pneumonia onset
I2	Readmission to intensive care
I3	In-unit procedure
I4	Intubation/reintubation
I5	Other
Emergency department	
E1	Readmission to ED within 48 h
E2	Time in ED greater than 6 h
E3	Other

### 2.5 Data collection process

Data were collected between February 1 and October 31, 2020, and included the clinical records of patients discharged between November 1, 2019, and July 31, 2020. This period was defined to ensure the eligibility of clinical records according to the established criteria, specifically a maximum interval of 2 months between discharge and review, thereby ensuring the integrity and legibility of the documentation.

The review team comprised clinically trained healthcare professionals, specifically, nurses and physicians with experience in hospital care. Prior to the formal data collection phase, all team members participated in a structured theoretical and practical training program to ensure full understanding of the GTT-PT methodology. The training sessions included familiarization with the tool modules and triggers, discussions of simulated cases, practical exercises, and orientation in using the data collection forms.

As a preparatory step, the team’s internal consistency was evaluated through an independent review of a sample set of clinical records by all reviewers. The objective was to guarantee a minimum inter-rater agreement of 80%, as recommended by the IHI, as an acceptable reliability standard for institutional multicenter implementation of the GTT (5).

**Figure 1:**
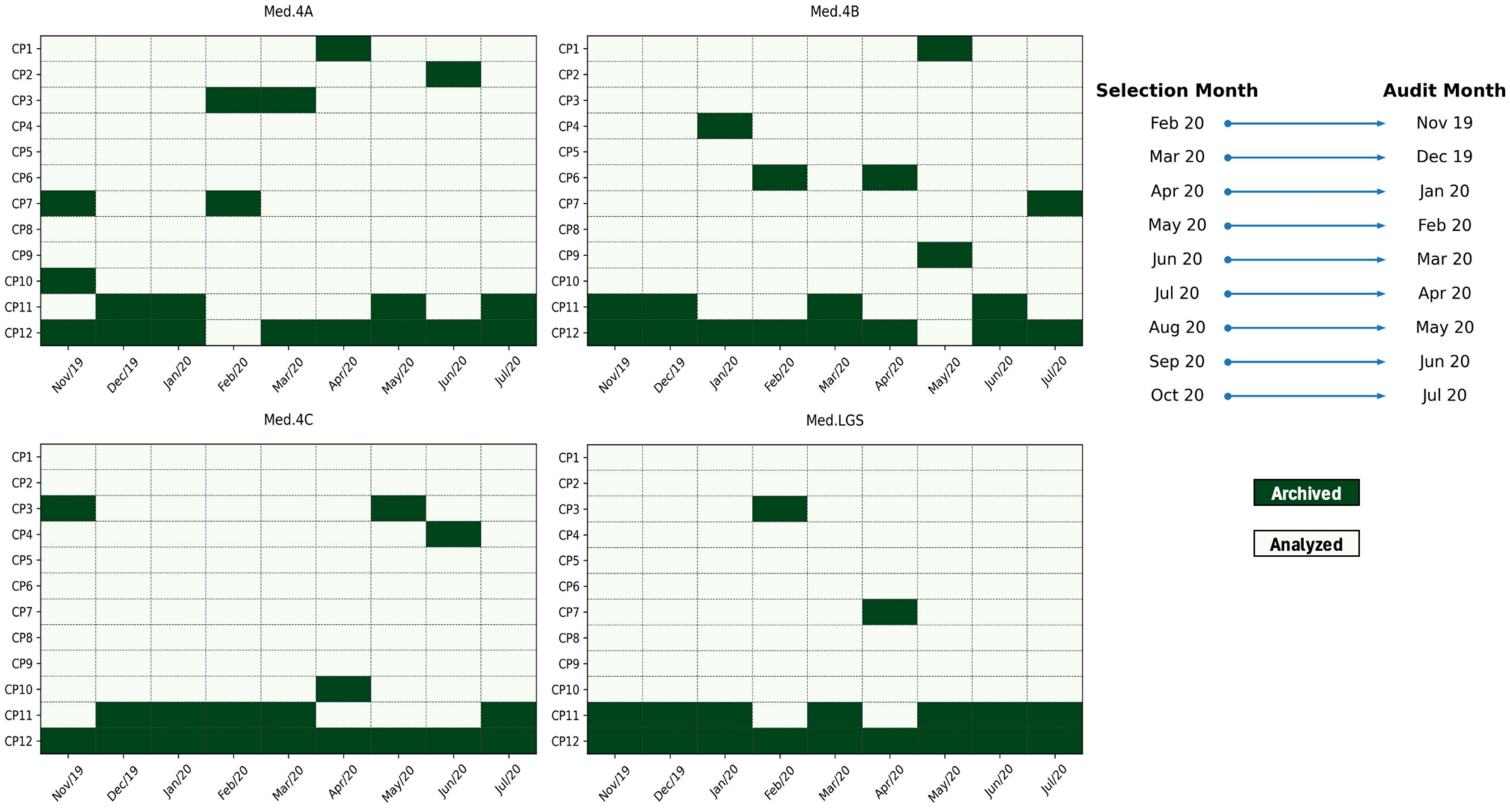
Representation of the process used for selecting and analyzing the included medical records.

Throughout the data collection period, each month, the same set of 10 randomly selected clinical records was independently reviewed by two nurses. At the end of each month, consensus meetings were held to discuss the findings, specifically, the presence of triggers and potential AEs. The confirmation or exclusion of each adverse event (AE) was based on consensus between the two reviewers, following the methodological guidelines of the IHI. In cases where consensus was not reached, the process was submitted for review by an independent physician, who acted as an arbitrator to ensure the consistency and validity of the final decision. Inter-rater reliability between the reviewers was subsequently analyzed using Cohen’s kappa coefficient, and the results are presented in the Results section. Each confirmed AE was classified according to the IHI severity scale (levels E to I). Situations in which the harm did not result from the provision of healthcare, or where documentation was insufficient to confirm causality, were excluded after joint deliberation. Figure 1 provides a schematic representation of the process used for selecting and analyzing the included medical records, 10 records reviewed per ward per month (4 wards × 9 months = 360 records).

### 2.6 Data analysis

Statistical analysis was performed using IBM SPSS Statistics version 27. Descriptive statistics were applied, including the calculation of absolute and relative frequencies, means, medians, standard deviations, and confidence intervals, according to the nature of each variable.

Multivariate binary logistic regression was used to analyze the association between sociodemographic and clinical variables and AE occurrence. This approach allowed estimation of the independent effect of each predictor variable on the likelihood of AE incidence, adjusting for potential confounders. The model included the variables age, sex, inpatient ward, length of stay, and presence of invasive devices, which were selected based on clinical relevance and preliminary descriptive analysis. Results are presented as odds ratio (OR) with 95% confidence intervals (95%CI).

Monthly randomization of clinical records was conducted using OpenEpi software, version 3.01, to ensure transparency, impartiality, and reproducibility in the selection process.

Microsoft Excel spreadsheets were used for preliminary data processing and the preparation of statistical tables.

Based on the data collected, the following patient safety indicators were calculated:

- Number of AEs per 100 admissions.
- Number of AEs per 1,000 hospitalization days.
- AE rate by category of harm, according to the IHI severity scale.
- Overall AE rate, based on the total number of reviewed cases.

These indicators allowed quantification and analysis of the frequency, distribution, and severity of AEs, contributing to a detailed assessment of patient safety.

### 2.7 Ethical considerations

This study was conducted in accordance with the principles outlined in the Declaration of Helsinki and the applicable ethical standards for health research. The study was approved by the CHUA Health Ethics Committee and Board of Directors on January 11, 2018.

As this was a retrospective observational study based solely on a review of clinical records with no direct contact with patients or collection of identifiable data, individual informed consent was waived. Prior to data collection, all medical records were coded to ensure data anonymization and confidentiality of patient information. Moreover, formal authorization was obtained from the IHI for the use of the GTT within the scope of its national adaptation and application as the GTT-PT.

## 3 Results

### 3.1 Sample characteristics

A random sample of 442 clinical records was selected from 2,287 clinical discharges recorded in the medical department, which comprised four wards with 108 records per ward. After applying the inclusion and exclusion criteria, 360 records (90 per ward) were audited and the remaining records were retained as substitutes. Each case was reviewed for an average of 27.4 min.

The average patient age was 75.4 years (range 36–96 years, SD = 11.7), with a slight prevalence of women (53%). The sample comprised 594 hospitalizations, with an average of 1.65 hospitalizations per patient (minimum 1, maximum 7); 214 patients (59.4%) had only one hospitalization each. The total number of hospitalization days was 9,642, with the lengths of stay ranging from 2 to 212 days per patient.

Most admissions (91%) occurred through the emergency service (ES). Admissions were primarily related to exacerbation of chronic disease, acute phase of a medical condition, or rehabilitation or occurred while awaiting social resolution. Most patients (91%) required at least one invasive medical device during hospitalization. Table 2 presents the sample characterization by ward.

**Table 2:** Sample characterization by ward.

No. and days of hospitalization	LG ward	4A ward	4B ward	4C ward
Mean length of stay in the past year	37.44	25.59	22.73	21.37
No. of patients with one hospitalization	44	51	55	64
No. of hospitalizations	176	145	145	128
Number of patients with invasive medical devices				
Urinary catheter	34	30	28	25
Peripheral or central venous catheter	82	81	81	87
Nasogastric tube	9	6	9	8
Others (e.g., paracentesis, intubation)	6	6	7	5

### 3.2 Trigger detection using the GTT-PT

During the review of the 360 clinical records in the sample conducted by four teams of previously trained primary reviewers, 1,541 triggers were identified. Inter-rater reliability among the reviewers was 92%, as measured by Cohen’s kappa coefficient, indicating a high level of agreement in the application and interpretation of the instrument.

The most frequently identified triggers included healthcare-associated infections, readmissions within 30 days, ES stays exceeding 6 h, patient falls, pressure ulcers, use of restraints, sleep disturbances with psychomotor agitation lasting more than 48 h, a two-fold increase in blood urea or creatinine from baseline, and impaired skin integrity.

Table 3 presents the distribution of triggers by module—general care, surgical care, medication/laboratory, intensive care, and emergency/urgent care—as well as by hospital ward. The general care module accounted for the highest number of identified triggers.

**Table 3:** Absolute number of triggers identified by ward.

Trigger class	Absolute no. of triggers by ward			
	LG ward	4A ward	4B ward	4C ward
Module – general care				
Blood transfusion or use of blood components	17	2	6	9
Emergency alert codes, cardiopulmonary resuscitation, or emergency team activation	10	9	1	4
Positive blood culture	5	16	3	5
X-ray or Doppler for suspected embolism or deep vein thrombosis	8	5	2	2
Rapid decrease of ≥ 25% in hemoglobin or hematocrit levels	0	1	1	5
Patient falls	19	11	16	11
Pressure ulcers	19	14	13	9
Readmission within 30 days	19	25	22	10
Use of restraints (mechanical/physical)	27	19	19	23
Healthcare-associated infections	38	38	24	26
In-hospital cardiac arrest	19	11	14	7
Transfer to higher level of care	6	13	3	7
Procedure-related complications	30	26	25	18
Sleep disturbance > 48 h	2	19	11	1
Skin lesions	14	27	32	24
Module – surgical care				
Mechanical ventilation > 24 h postoperatively	1	2	4	2
Intraoperative administration of adrenaline, noradrenaline, naloxone, or flumazenil	1	1	2	0
Intraoperative injury, repair, or removal of an organ	0	1	2	0
Intraoperative complications	0	1	2	0
Module – laboratory parameters and medication				
*Clostridium difficile* – positive culture	6	3	1	1
International normalized ratio (INR) > 6	3	1	2	1
Blood glucose < 50 mg/dL	7	1	3	4
Serum urea or creatinine > 2x baseline	34	18	9	27
Administration of vitamin K	2	0	2	3
Administration of flumazenil or naloxone	6	4	4	5
Antiemetic administration (for uncontrolled vomiting)	15	5	18	18
Administration of antihistamines (clemastine, diphenhydramine, Benadryl)	3	4	1	3
Sudden medication discontinuation	11	9	16	4
Overdose – sedation/hypotension	3	5	1	4
Administration of as-needed medications (pain/blood pressure) > 48 h	10	49	36	18
Module – Intensive care				
Onset of pneumonia	2	4	2	3
Readmission to intensive care unit (ICU)	2	1	1	1
ICU procedures	0	5	1	4
Intubation/reintubation	0	4	1	4
Module – urgent/emergency service care				
ES readmission within 48 h (same cause)	20	11	16	17
ES stay > 6 h	35	46	65	75
Total	394	411	381	355

### 3.3 AE identification using the GTT-PT

A total of 718 AEs were identified in the 360 audited clinical records comprising the final sample. Among these, 564 AEs (78.6%) occurred during hospitalization, while 154 (21.4%) were already present at the time of hospital admission.

The most prevalent AEs during hospitalization included the use of restraints (*n* = 84), readmission within 30 days (*n* = 81), a two-fold increase in serum urea or creatinine levels (*n* = 43), patient falls (*n* = 38), and pressure ulcers (*n* = 33). Additional events were associated with healthcare-associated infections, such as hospital-acquired pneumonia, urinary tract infections, phlebitis, and septicemia, as well as adverse drug reactions, including hypoglycemia, uncontrolled pain, and allergic reactions.

Among the AEs present at admission, the most frequent were healthcare-associated infections in patients with urinary catheters in the ES (*n* = 28), aspiration pneumonia (*n* = 24), and pressure ulcers (*n* = 25).

Table 4 provides a detailed breakdown of AEs by category, hospital ward, and time of detection (hospitalization vs. admission).

**Table 4:** Absolute number of AEs by ward and cause, categorized as originating during hospitalization or present on admission.

AE types	Absolute no. of AEs by ward				Total no. in the department	% AEs in the department
	LG ward	4A ward	4B ward	4C ward		
AEs identified during hospitalization						
Patient falls	11	7	12	8	38	6.8
Pressure ulcer	13	8	7	5	33	5.9
Traumatic wound/skin detachment	0	6	12	6	24	4.4
Diaper rash/erythema	3	27	3	3	36	6.4
Readmission within 30 days	18	21	22	20	81	14.5
Use of restraints (mechanical)	27	19	19	19	84	14.9
Blood glucose < 50 mg/dL	3	1	1	1	6	1.1
Serum urea or creatinine > 2x baseline	15	12	8	8	43	7.7
Antiemetic administration (for uncontrolled vomiting)	3	2	2	2	9	1.6
In-hospital aspiration pneumonia	3	4	8	8	23	4.1
Puncture site infection/phlebitis	5	4	7	7	23	4.1
Urinary tract infection (post-catheterization), no agent identified	7	8	7	7	29	5.2
Healthcare-associated pneumonia	5	6	5	5	21	3.8
*Escherichia coli* or *Pseudomonas* in fluids	6	5	7	4	22	3.9
*Candida parapsilosis* in urine	2	2	2	2	8	1.4
Methicillin-resistant *Staphylococcus aureus* (MRSA) in urine, urethral exudate, sputum, or blood	3	3	7	7	20	3.5
*Enterobacter cloacae* or *Morganella morganii* in urine	6	3	2	2	13	2.3
Conjunctivitis	7	5	3	3	18	3.2
Septicemia	6	4	4	2	16	2.8
*Clostridium difficile* – positive culture	2	2	2	0	6	1.1
Allergic reaction (with documented allergy)	0	0	1	0	1	0.2
Uncontrolled pain > 72 h	0	0	5	5	10	1.8
Total:	145	149	146	124	564	100%
AEs identified on hospital admission						
Aspiration pneumonia (institutionalized patient)	7	7	7	3	24	16.2
Healthcare-associated infection in a patient catheterized in the emergency service	9	8	4	7	28	18.3
Overdose – sedation/hypotension	0	0	0	3	3	2.2
Septicemia	5	2	0	3	10	7
Patient falls	8	6	4	3	21	14.7
Pressure ulcers	9	6	6	4	25	17.4
*Enterobacter cloacae* in urine	2	2	2	3	9	6.4
MRSA in urine or blood	1	2	2	2	7	5
*Escherichia coli*, *Candida parapsilosis*, or *pseudomonas*	4	7	4	2	17	12
Total:	45	40	29	29	144	100%

**Figure 2:**
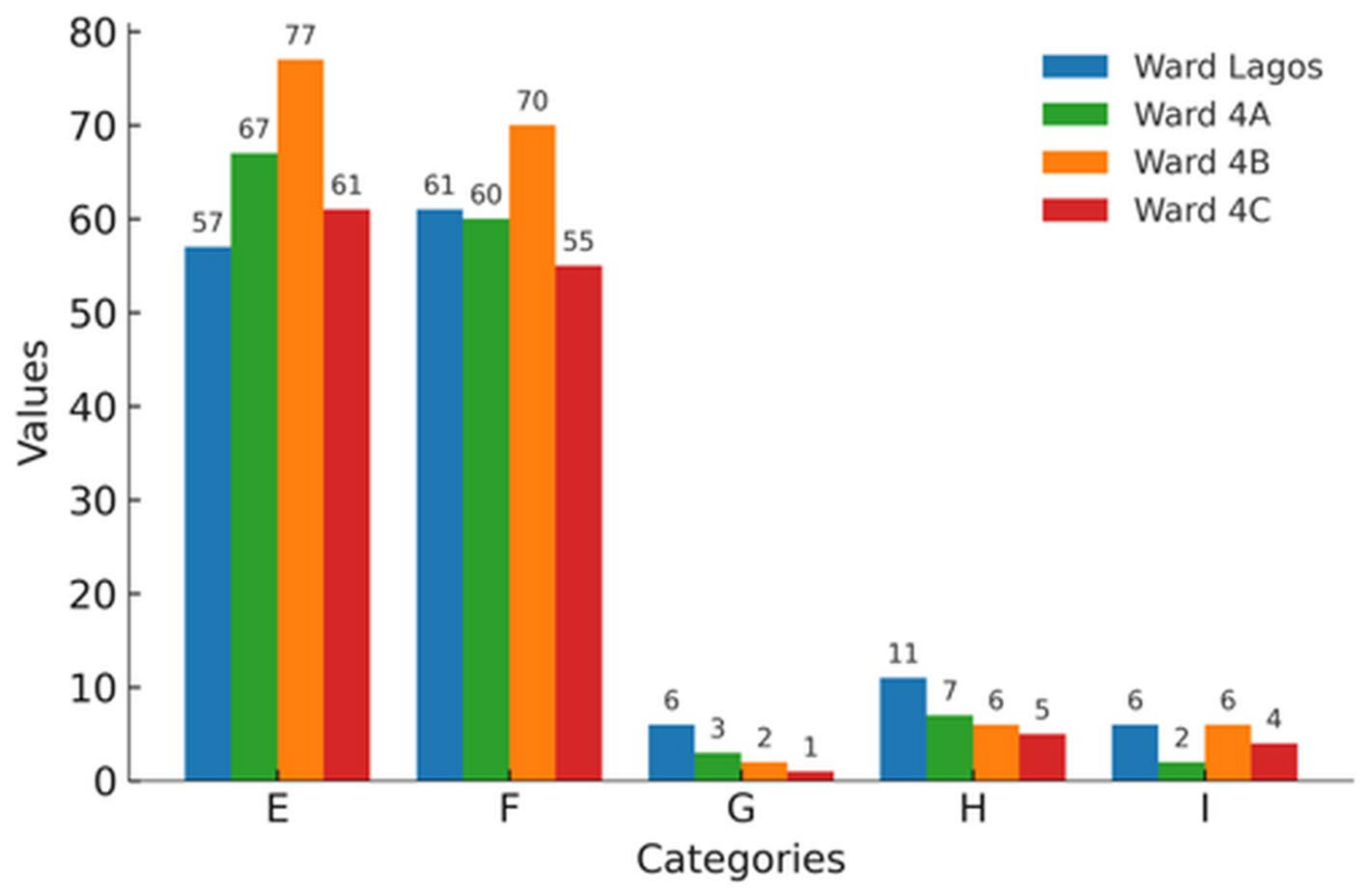
Absolute frequency of AEs by severity and ward.

The severity of the 564 AEs that occurred during hospitalization was measured using the adapted IHI scale, ranging from categories E to I. Figure 2 illustrates the distribution of AEs by harm level and hospital ward.

Most AEs were classified as level E (temporary harm requiring intervention) or level F (temporary harm requiring prolonged hospitalization), totaling 426 cases that occurred during hospitalization (75.5%).

Safety indicators related to AE frequency were calculated using the data collected using the GTT-PT, as Table 5 shows.

**Table 5:** Safety indicators collected using the GTT-PT.

Indicator	Ward Lagos	Ward 4A	Ward 4B	Ward 4C	In the department
AEs per 1,000 patient days	78.7	120.6	112.6	88.4	100.1
AEs per 100 hospitalizations	161	165.6	162.2	137.8	156.7
% of hospitalizations with AEs	73%	82%	80%	64.4%	74.9%
% of hospitalizations with one AE	10.3%	9.2%	11.8%	7.8%	9.8%

**Figure 3:**
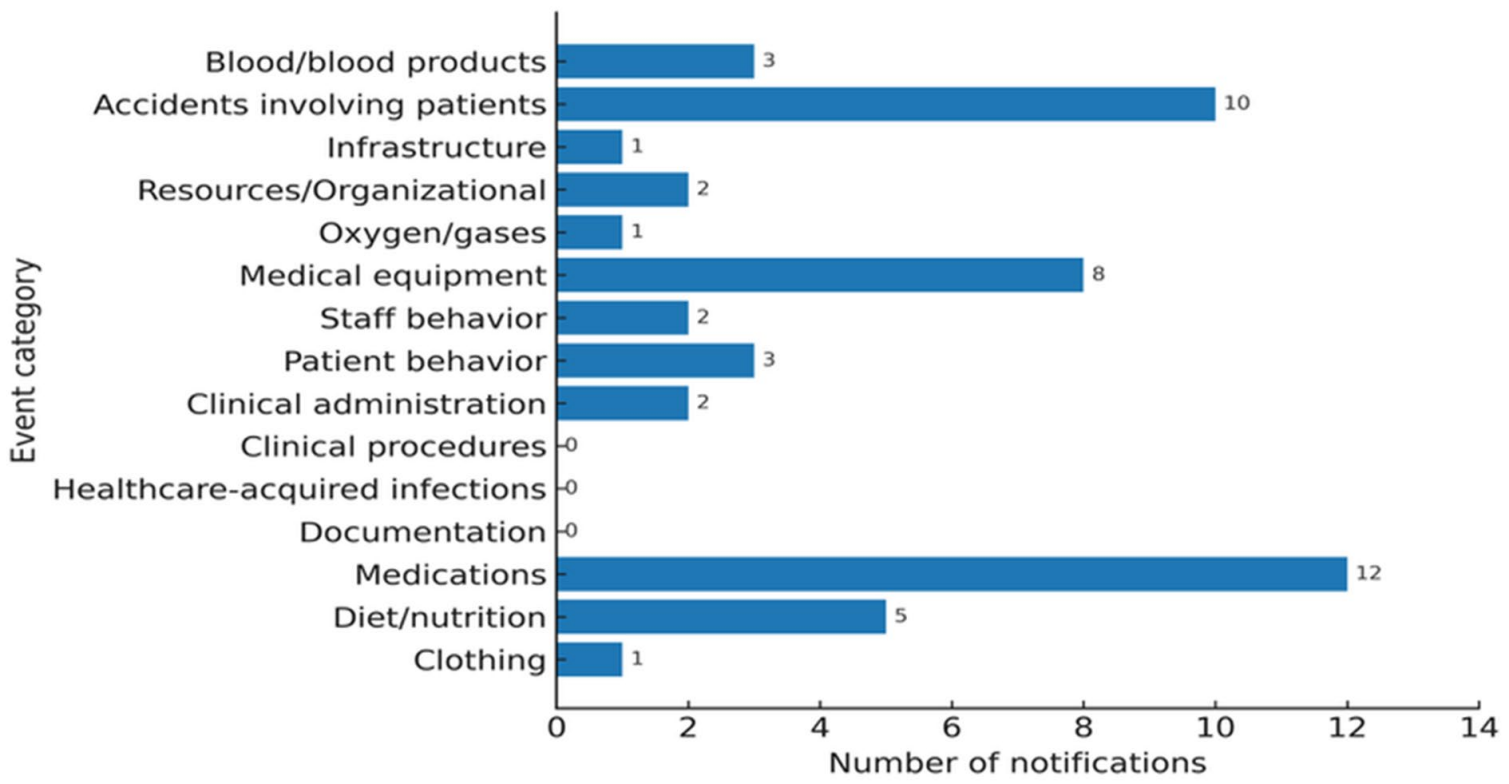
Distribution of voluntary reports by event category.

During the observation period, 564 adverse events were identified through the application of the GTT-PT, while the institutional voluntary reporting system registered 50 events, representing an eleven fold difference in detection capacity between the two methods (Figure 3).

Data from the four wards within the Department of Medicine were examined using binary logistic regression to identify factors associated with the occurrence of AEs. All models met the statistical assumptions for the absence of multicollinearity (variance inflation factor < 5).

Each model included nine independent variables, covering sociodemographic characteristics (age and sex), clinical history (number of hospitalizations and length of stay), and presence of invasive devices (peripheral or central venous catheter, urinary catheter, nasogastric tube [NGT], paracentesis, or endotracheal tube).

The logistic regression models were developed using the stepwise forward likelihood ratio method (Forward LR), which allows for the progressive inclusion of variables based on their statistical contribution to the model. Candidate variables were previously selected based on clinical relevance, evidence described in the literature, and their behavior in exploratory univariate analyses. Entry and removal criteria were applied as defined by *p* < 0.05 and *p* > 0.10, respectively, ensuring that only predictors with statistically significant contributions were retained in the final model. This approach ensured control of potential confounding factors by keeping relevant demographic and clinical variables in the model and contributed to increasing the robustness and internal validity of the results.

Three of the four wards (LG, 4A, and 4C) presented statistically significant models (p < 0.05) with good fit (Hosmer–Lemeshow test, *p* > 0.80) and Nagelkerke R^2^ values ranging from 0.35 to 0.45. The number of hospitalization days was a significant predictor of AEs in these wards, with OR ranging from 1.011 to 1.173. In the LG ward, the presence of an NGT was statistically significant. In the 4B ward, the overall model was not significant; however, the variable “length of stay” showed significance when analyzed individually (*p* = 0.038) (Table 6).

**Table 6:** Binary logistic regression results by ward (final model variables).

Ward	Predictor variable	Exp(B)	gl	OR (95% CI)	*p*-value
LG ward	Hospitalization days	0.011	1	1.011 (1.003–1.019)	0.008
LG ward	Nasogastric tube present	1.902	1	6.693 (1.223–36.635)	0.028
4A ward	Hospitalization days	0.160	1	1.173 (1.059–1.300)	0.002
4B ward	Hospitalization days	0.132	1	1.141 (1.007–1.293)	0.038
4C ward	Hospitalization days	0.138	1	1.148 (1.019–1.294)	0.024

To assess the robustness of the logistic regression models, two complementary sensitivity analyses were performed. First, a global influence analysis was conducted, applying the criterion of Cook’s distance > 1 to identify observations with an excessive impact on the model. Four cases were removed (1.1% of the total sample), without any substantial changes being observed in the direction or significance of the main predictors, confirming that the model was not influenced by outlier observations. Additionally, the presence of influential observations within each ward was assessed, and no cases with relevant impact on the respective submodels were identified. Next, a sensitivity analysis by subgroup exclusion (leave-one-ward-out) was performed, re-running the models after alternately removing each ward. This procedure confirmed that the main associations—namely the length of hospital stay and, specifically in the LG ward, the use of a nasogastric tube—maintained similar direction and significance, confirming the stability and robustness of the results.

## 4 Discussion

The results of this study reveal a high incidence of adverse events (AEs) in internal medicine wards, consistent with international evidence indicating that up to 10% of hospitalizations are associated with some type of AE, a substantial proportion of which is potentially preventable (1–3). When comparing the results of this study with international data, various contextual aspects must be considered for a critical interpretation of the observed differences. The incidence of AEs identified in this study (approximately 160 AEs per 100 hospitalizations, or 78.7–120.6 per 1,000 patient-days) was slightly higher than that reported by Grossmann et al. (12) in Switzerland (110 AEs per 1,000 patient-days) and by Hibbert et al. (7) in Australia (95 AEs per 1,000 patient-days). These variations may reflect the greater clinical complexity and multimorbidity of the Portuguese hospitalized population, as well as the systematic use of the GTT-PT, which allows for the detection of less severe events that may not be captured by studies using simplified tools (24). Furthermore, differences in AE rates between studies may be related to variations in institutional safety culture, the quality of documentation, and the maturity level of patient safety governance (25). These results reinforce that comparisons between studies should consider contextual differences in patient profiles, safety culture, and methodological sensitivity, highlighting that the prevalence of AEs should be viewed not only as a performance indicator but also as a measure of the institution’s capacity for learning. Multivariate analysis showed that the length of hospital stay was statistically significantly associated with the occurrence of AEs in the LG, 4A, and 4C wards, indicating that longer hospitalizations are associated with greater exposure to cumulative iatrogenic risk factors, and not necessarily with a direct causal relationship. In the LG ward, the presence of a nasogastric tube (NGT) was also associated with a higher probability of AEs occurring (OR = 6.693), which may reflect greater clinical severity of the patients and characteristics of care practices related to the use of NGTs. Although the overall model for ward 4B was not statistically significant, the association between length of stay and the occurrence of AEs remained significant when analyzed separately (*p* = 0.038), reinforcing the consistency of this association across different wards.

Regarding detection methods, the combined use of the GTT-PT and voluntary reporting has proven to be a valuable complementary strategy. The GTT-PT demonstrated superior sensitivity, particularly in identifying adverse drug reactions, healthcare-associated infections, and readmissions, aligning with previous studies that show trigger tools can detect up to 10 times more AEs than reactive reporting systems (7, 24). Conversely, the well-documented limitations of voluntary reporting were confirmed, notably chronic underreporting due to institutional barriers, fear of punitive consequences, and lack of feedback (5). Evidence from Nakatani et al. indicates that educational interventions and structural changes can reduce AE incidence (from 39.9 to 18.4 per 1,000 cases) while increasing staff engagement in safety monitoring (25).

The complementarity of the GTT-PT and voluntary reporting offers a broader and more robust surveillance approach, as each method captures distinct AE subsets. Studies by Grossmann et al. and Wu et al. highlighted the added value of combining these systems in complex contexts, such as obstetrics and geriatrics, while Kandaswamy et al. (15) emphasized the importance of continuous monitoring and real-time AE analysis for the dynamic adaptation of safety strategies (12, 13). Nevertheless, comparative studies have demonstrated minimal overlap between the two methods, underscoring the limitation of relying on a single tool for a comprehensive safety assessment (15).

These findings reinforce the need for integrated surveillance systems supported by nonpunitive institutional policies. Beyond methodological complementarity, sustainable improvements in patient safety require investment in advanced digital technologies, continuous professional training, structured feedback mechanisms, and strong organizational commitments. Importantly, the quantification and stratification of AEs by type, ward, and severity revealed that although most events were less severe, a substantial proportion caused significant harm. This underscores the urgency of implementing robust preventive and responsive strategies during hospitalization.

Future efforts should prioritize hybrid surveillance models that integrate retrospective trigger-based reviews with real-time digital monitoring. This approach aims to enhance sensitivity and foster sustainable improvements in hospital safety practices. In this regard, the combined use of the GTT-PT and voluntary reporting systems has emerged as a promising complementary strategy. While the GTT offers a robust retrospective evaluation based on clinical records, voluntary reporting captures adverse events (AEs) directly observed by healthcare professionals during care procedures. By incorporating both methods, AE detection is enhanced, providing a more comprehensive view of hospital safety and enabling more consistent comparisons across various contexts and institutions (7, 26, 27).

However, this complementarity has several limitations. Comparative studies have shown minimal overlap between AEs detected by the two methods, indicating that each strategy captures distinct subsets of AEs. This highlights the limitation of relying on a single method for a comprehensive evaluation of patient safety (28). These indicators facilitate the quantification and analysis of AE incidence and categorization by type, ward, and severity, contributing to a comprehensive evaluation of patient safety in hospital settings. Although most identified AEs were less severe, a significant proportion of cases involved substantial harm, underscoring the importance of robust precautionary and responsive strategies during hospitalization. It is crucial to emphasize that, due to the observational and retrospective nature of this study, the relationships identified between the variables represent statistical associations and do not imply direct causality. Therefore, the results should be interpreted as indicative trends, highlighting factors potentially associated with the risk of adverse events and opportunities for improvement in patient safety processes.

### 4.1 Strengths and limitations

One of the primary strengths of this study was the integration of two complementary methods, the GTT-PT and the voluntary reporting system, which facilitated a more thorough analysis of AE incidence within a hospital setting. The systematic application of the GTT-PT ensured high sensitivity in detecting AEs, including those not typically identified by conventional methods. However, this study does have certain limitations. A retrospective review of clinical records depends on data quality, which can lead to AE underestimation in cases of incomplete or ambiguous records. Beyond these limitations, potential biases must also be acknowledged. Selection bias may have occurred because only complete and legible medical records were included, potentially excluding more complex or acute cases. Information bias might have resulted from inconsistencies or omissions in clinical documentation, leading to possible underdetection of AEs. Additionally, observer bias could have arisen from manual record review, although this was minimized through standardized reviewer training, high inter-rater reliability (*κ* = 0.92), and consensus validation. Furthermore, the models were not designed for causal inference and did not include medication-related variables (e.g., polypharmacy, drug–drug interactions), as these data were inconsistently documented in retrospective records. Future prospective studies should explore these factors to clarify potential causal mechanisms underlying adverse events. Moreover, the application of the GTT-PT is resource-intensive and requires trained personnel and significant time, which may limit its use in routine hospital audits. Finally, the study was conducted at a single hospital in southern Portugal, which restricts the generalizability of the findings to other institutional or geographic contexts.

### 4.2 Future research directions

Future studies could expand on this research by including multicenter and nationally representative samples to validate the applicability of the GTT-PT across diverse healthcare settings. It is also important to evaluate the long-term effects of regular GTT-PT on AE reduction. In addition, integrating digital technologies and artificial intelligence into the automated analysis of clinical records could improve efficiency, reduce the burden on reviewers, and allow for continuous surveillance. Future studies should investigate how educational initiatives and institutional campaigns influence staff adherence to voluntary reporting systems.

## 5 Conclusion

This study confirmed the high incidence of AEs among patients hospitalized in internal medicine wards, particularly in populations with multiple morbidities. The implementation of the GTT-PT was demonstrated to be a robust and sensitive method, identifying a substantially greater number of AEs than the institutional voluntary reporting system. These results highlight the complementary nature of the two approaches, each capturing different types of AEs and contributing to a more comprehensive understanding of hospital safety.

Multivariate analysis identified the number of days of hospitalization as the primary predictor of AEs, indicating that a longer hospital stay increases patient exposure to potential iatrogenic harm. Furthermore, the presence of invasive devices—specifically NGT—was associated with a higher risk of AEs, reinforcing the need for stringent clinical protocols in the use of such devices.

Although this study is limited by its retrospective design and single-center application of GTT-PT, the findings offer valuable insights to guide institutional strategies aimed at preventing, detecting, and mitigating AEs. The development of integrated patient safety surveillance systems—anchored in real-world, context-specific data—can promote a culture of organizational learning and continuous improvement.

In practical terms, the findings of this study have significant implications for hospital management, patient safety governance, and workforce development. The identification of prolonged hospitalization and nasogastric tube use as key predictors of adverse events highlights the necessity of enhancing multidisciplinary monitoring of high-risk patients and implementing targeted training programs on device management and infection prevention. At the managerial level, integrating the GTT-PT with voluntary reporting systems can augment institutional learning by facilitating data-driven decision-making, resource prioritization, and the formulation of evidence-based safety policies. Furthermore, fostering a culture of openness and shared accountability can enhance healthcare professionals’ engagement and promote sustainable safety improvements.

Future research should extend this approach to national and international multicenter settings, explore technological solutions for automating trigger identification, and facilitate the routine use of the GTT-PT. In parallel, the implementation of training programs and institutional awareness campaigns is essential for strengthening healthcare professionals’ engagement with voluntary reporting systems, ultimately contributing to the creation of safer, more sustainable, and patient-centered hospital environments.

## Data Availability

The original contributions presented in the study are included in the article/supplementary material, further inquiries can be directed to the corresponding author.

## References

[1] De Vries EN Ramrattan MA Smorenburg SM Gouma DJ Boermeester MA. The incidence and nature of in-hospital adverse events: a systematic review. Qual Saf Health Care. (2008) 17:216–23. doi: 10.1136/qshc.2007.023622, 18519629 PMC2569153

[2] Kjellberg J Wolf RT Kruse M Rasmussen SR Vestergaard J Nielsen KJ . Costs associated with adverse events among acute patients. BMC Health Serv Res. (2017) 17:651. doi: 10.1186/s12913-017-2605-5, 28903748 PMC5598051

[3] Sauro KM Machan M Whalen-Browne L Owen V Wu G Stelfox HT. Evolving factors in hospital safety: a systematic review and meta-analysis of hospital adverse events. J Patient Saf. (2021) 17:e1285–95. doi: 10.1097/PTS.0000000000000889, 34469915

[4] Schildmeijer KGI Unbeck M Ekstedt M Lindblad M Nilsson L. Adverse events in patients in home healthcare: a retrospective record review using trigger tool methodology. BMJ Open. (2018) 8:e019267. doi: 10.1136/bmjopen-2017-019267, 29301764 PMC5781156

[5] Griffin FA Resar RK. IHI global trigger tool for measuring adverse events. Cambridge: Institute for Healthcare Improvement (2009).

[6] Hanskamp-Sebregts M Zegers M Vincent C van Gurp PJ de Vet HCW Wollersheim H. Measurement of patient safety: a systematic review of the reliability and validity of adverse event detection with record review. BMJ Open. (2016) 6:e011078. doi: 10.1136/bmjopen-2016-011078, 27550650 PMC5013509

[7] Hibbert PD Molloy CJ Schultz TJ Carson-Stevens A Braithwaite J. Comparing rates of adverse events detected in incident reporting and the global trigger tool: a systematic review. Int J Qual Health Care. (2023) 35:mzad056. doi: 10.1093/intqhc/mzad056, 37440353 PMC10367579

[8] Mevik K Hansen TE Deilkås EC Ringdal AM Vonen B. Is a modified global trigger tool method using automatic trigger identification valid when measuring adverse events? A. Int J Qual Health Care. (2019) 31:535–40. doi: 10.1093/intqhc/mzy210 30295829

[9] Pierdevara L Porcel-Gálvez AM Ferreira da Silva AM Barrientos Trigo S Eiras M. Translation, cross-cultural adaptation, and measurement properties of the Portuguese version of the global trigger tool for adverse events. Ther Clin Risk Manag. (2020) 16:1175–83. doi: 10.2147/TCRM.S282294, 33299318 PMC7721282

[10] Johnston MC Crilly M Black C Prescott GJ Mercer SW. Defining and measuring multimorbidity: a systematic review of systematic reviews. Eur J Pub Health. (2019) 29:182–9. doi: 10.1093/eurpub/cky098, 29878097

[11] Hu Q Qin Z Zhan M Chen Z Wu B Xu T. Validating the Chinese geriatric trigger tool and analyzing adverse drug event associated risk factors in elderly Chinese patients: a retrospective review. PLoS One. (2020) 15:e0232095. doi: 10.1371/journal.pone.0232095, 32343726 PMC7188209

[12] Grossmann N Gratwohl F Musy SN Nielen NM Donzé J Simon M. Describing adverse events in medical inpatients using the global trigger tool. Swiss Med Wkly. (2019) 149:w20149. doi: 10.4414/smw.2019.20149, 31707720

[13] Thomas EJ Brennan TA. Incidence and types of preventable adverse events in elderly patients: population based review of medical records. BMJ. (2000) 320:741–4. doi: 10.1136/bmj.320.7237.741, 10720355 PMC27315

[14] Blayney MC Reed MJ Masterson JA Anand A Bouamrane MM Fleuriot J . Multimorbidity and adverse outcomes following emergency department attendance: population based cohort study. BMJ Med. (2024) 3:e000731. doi: 10.1136/bmjmed-2023-000731, 39184567 PMC11344864

[15] Kandaswamy S Josephson CD Rollins MR Jones J Zerra P Goel R . Development and evaluation of trigger tools to identify pediatric blood management errors. Blood Transfus. (2024) 22:484–91. doi: 10.2450/BloodTransfus.606, 38557324 PMC11576144

[16] Oweidat I Al-Mugheed K Alsenany SA Abdelaliem SMF Alzoubi MM. Awareness of reporting practices and barriers to incident reporting among nurses. BMC Nurs. (2023) 22:231. doi: 10.1186/s12912-023-01376-9, 37400810 PMC10318788

[17] Wu F Wang X Chen S Li H Xie H. Nurses’ adverse event reporting attitudes and related factors: a cross-sectional study in maternal and child specialized hospitals in China. Front Public Health. (2024) 12:1434387. doi: 10.3389/fpubh.2024.1434387, 39712313 PMC11659206

[18] Almansour H. Barriers preventing the reporting of incidents and near misses among healthcare professionals. J Health Manag. (2024) 26:78–84. doi: 10.1177/09720634231167031

[19] Dan X He YL Huang Y Ren JH Wang DQ Yin RT . Construction and evaluation of a cloud follow-up platform for gynecological patients receiving chemotherapy. BMC Health Serv Res. (2024) 24:116. doi: 10.1186/s12913-024-10597-w, 38254152 PMC10802037

[20] World Health Organization. Patient safety incident reporting and learning systems: Technical report and guidance. Geneva: World Health Organization (2020).

[21] Fekadu G Muir R Tobiano G Ireland MJ Engidaw MT Marshall AP. Patient safety incident reporting systems and reporting practices in African healthcare organisations: a systematic review and meta-analysis. BMJ Open Qual. (2025) 14:e003202. doi: 10.1136/bmjoq-2024-003202, 40011060 PMC11865795

[22] Hibbert PD Stewart S Wiles LK Braithwaite J Runciman WB Thomas MJW. Improving patient safety governance and systems through learning from successes and failures: qualitative surveys and interviews with international experts. Int J Qual Health Care. (2023) 35:01. doi: 10.1093/intqhc/mzad088. 37978851; PMCID: PMC10656601, 37978851 PMC10656601

[23] von Elm E Altman DG Egger M Pocock SJ Gøtzsche PC Vandenbroucke JP . The strengthening the reporting of observational studies in epidemiology (STROBE) statement: guidelines for reporting observational studies. PLoS Med. (2007) 4:e296. doi: 10.1371/journal.pmed.0040296, 17941714 PMC2020495

[24] Classen DC Resar R Griffin F Federico F Frankel T Kimmel N . ‘Global trigger tool’ shows that adverse events in hospitals may be ten times greater than previously measured. Health Aff. (2011) 30:581–9. doi: 10.1377/hlthaff.2011.0190, 21471476

[25] Nakatani K Nakagami-Yamaguchi E Hagawa N Tokuwame A Ehara S Nishimura T . Evaluation of a new patient safety educational programme to reduce adverse events by encouraging staff to speak up: application of the trigger tool methodology. BMJ Open Qual. (2024) 13:e002162. doi: 10.1136/bmjoq-2022-002162, 38212131 PMC10806700

[26] Samal L Khasnabish S Foskett C Zigmont K Faxvaag A Chang F . Comparison of a voluntary safety reporting system to a global trigger tool for identifying adverse events in an oncology population. J Patient Saf. (2022) 18:611–6. doi: 10.1097/PTS.0000000000001050, 35858480 PMC9391281

[27] Sansone V Paduano G D’Emma MR Pavia M. Assessment of the occurrence of adverse events through the global trigger tool in a university hospital in Italy. Sci Rep. (2025) 15:23973. doi: 10.1038/s41598-025-08617-8, 40615481 PMC12227586

[28] Moraes SM Ferrari TCA Figueiredo NMP Almeida TNC Sampaio CCL Andrade YCP . Assessment of the reliability of the IHI global trigger tool: new perspectives from a Brazilian study. Int J Qual Health Care. (2021) 33:mzab039. doi: 10.1093/intqhc/mzab039, 33676370

